# The Frequency of Smoking and Common Factors Leading to Continuation of Smoking among Health Care Providers in Tertiary Care Hospitals of Karachi

**DOI:** 10.5539/gjhs.v6n3p227

**Published:** 2014-03-30

**Authors:** Muhammad Shahzeb Khan, Faizan Imran Bawany, Muhammad Umer Ahmed, Mehwish Hussain, Noreen Maqbool Bukhari, Nighat Nisar, Maham Khan, Ahmed Raheem, Mohammad Hussham Arshad

**Affiliations:** 1 Dow Medical College, Dow University of Health Sciences, Karachi, Pakistan; 2 Ziauddin Medical University, Karachi, Pakistan; 3 Community Medicine Department, Dow University of Health Sciences, Karachi, Pakistan; 4 Aga Khan University Hospital, Karachi, Pakistan

**Keywords:** smoking, healthcare providers, tertiary care hospitals, factors

## Abstract

**Background:**

The primary objective of the study was to find out the frequency of tobacco smoking among health care providers in tertiary care hospitals of Karachi. The secondary objective was to identify the common factors responsible for the continuation of smoking.

**Method:**

This cross sectional study was conducted in the wards and out-patient departments of three selected tertiary hospitals of Karachi. A total of 180 health care providers were enrolled in the study using proportionate stratified sampling. Postgraduate students, house officers and trainees were excluded from the study. Data were collected from randomly selected health care providers using survey methodology. SPSS v. 20.0 was used to enter and analyze the data.

**Results:**

Fifty two participants out of 180 were smokers for past one year (28.9%). Among them, 21 (11.7%) smoked more than 5 cigarettes per day. Twenty smokers (11.1%) were found to smoke due to peer influence. It was found that those who were influenced by their peers were 8.33 times more prone to be addicted to smoking than those who were less influenced. Similarly, the likelihood of addiction increased up to 76.9% with the lack of incentives.

**Conclusion:**

Our results clearly indicate that a large number of health care providers smoke which should be a serious concern. Hence our health agencies should take immediate action in order to curtail the heaving burden of smoking and its related health consequences.

## 1. Introduction

Cigarette smoking is the largest preventable risk factor for morbidity and mortality in developed countries, where at least one in four adults smoke cigarettes (Thomson, Chaudhuri, & Livingston, 2004). Situation in the developing countries is even worse. It is estimated that by 2030 the developing world is expected to have 7 million deaths annually from tobacco use (Abdullah & Husten, 2004). Unfortunately, Pakistan is one of those countries where despite escalating knowledge and awareness programs, cigarette smoking is ever rising. With the smoking epidemic, the role of health care providers is most crucial in lowering down the smoking rates among the masses & thereby preventing many avoidable diseases.

Most ironical fact is that healthcare providers too have tendency to smoke. This is a global issue. Health Care Providers all over the world have been identified to be involved in smoking, at least to some extent. An American study (Serna, Bialuos, Sinha, Yang, & Wewers, 2010) indicates that in 2006/2007, Licensed Practical Nurses had the highest prevalence of tobacco smoking (20.55%) followed by respiratory therapists (19.28%). Physicians had a prevalence of 2.31%, dentists (3.01%), pharmacists (3.25%), and Registered Nurses (10.73%). The overall prevalence of smoking among health care providers was 9.85% (Serna et al.,2010). Similarly, a study from China (Yan et al.,2008) identified 20.8% of health care providers as current smokers. Smoking among physicians was very high (35.7%) according to this study and 59.7% of the respondents believed that inadequate knowledge was responsible for their continuation of smoking.

There are very few local studies addressing this problem. Little work has been done on a large scale to find the prevalence of smoking among health care providers and the significant factor responsible for their continuation of smoking besides its hazardous effects. A recent study from Lahore (Malick et al.,2010) conducted at Mayo hospital found out the frequency of smoking among doctors to be 37.18% and in paramedical staff to be 35.74%. Most of them initiated smoking due to the influence of friends. Majority of doctors and paramedics found smoking as relaxing/addicting and was the main reason they couldn’t quit. Main factors responsible for continuation of smoking were addiction (Doctors 38%, Paramedics 42%), lack of will power (Doctors 21%, Paramedics 27%) and lack of incentive (Doctors 24%, Paramedics 12%). On the other hand, among general practitioners, 36% were found to be cigarette smokers, who consumed 12.48 cigarettes per day and had 18.76 average years of smoking. Half of them had been smoking for more than 20 years (Nawaz & Naqvi, 2010).

Physicians who smoke are less likely to advise patients to quit smoking. Also, it is less expected from them to assess patient’s will to refrain from smoking (Asfar, Al-Ali, Ward, Vander Weg, & Maziak, 2011). In addition to all the smoking related health hazards that health care providers are exposed to, they are also not able to counsel their patients effectively. It is evident that if health care providers themselves smoke, they cannot educate masses regarding smoking cessation.

There is a need of a large scale study involving more than one tertiary care hospitals to find out the frequency of smoking among health care providers (both doctors & paramedics) and to appraise the reasons behind their continuation of smoking despite their medical edification. This study will help to identify factors responsible for continuation of smoking so that recommendations can be made for its cessation among health care providers and thereafter the community. The primary objective of the study was to find out the frequency of tobacco smoking among health care providers in tertiary care hospitals of Karachi. The secondary objective was to identify the common factors responsible for the continuation of smoking among health care providers.

## 2. Methods

This cross sectional study was conducted in wards and out-patient departments of three selected tertiary hospitals of Karachi namely Civil Hospital, PNS Shifa and Liaquat National Hospital. A sample size of 163 was calculated at 95% confidence interval by keeping the smallest frequency of responsible factors that was lack of incentive among paramedics as 12% (Malick et al.,2010). So a total of 180 health care providers were enrolled in the study using proportionate stratified sampling. Postgraduate students, house officers and trainees were excluded from the study. Doctors and paramedics (nurses and technicians) of either gender who were working in tertiary care hospitals were only included in the study. However, doctors and paramedics who were working in hospitals where beds were less than 200 were also excluded from the study.

In Karachi there are 110 private and public hospitals in a ratio of 2:1. So 2 private and 1 public hospital were selected to generalize our findings accurately. Proportionate sample was taken from each hospital. Doctors and paramedical staff were listed according to their hospital identity number provided by the hospitals to form sampling frame. Total number of doctors and paramedical staff at civil hospital were 604 and 1850 respectively and their proportionate sample calculated was 32 and 96 respectively. Therefore randomly we selected first doctor and paramedical staff by random number table and moved down the column selecting appropriate numbers that identified 32 doctors and 96 paramedical staff. Similarly for PNS Shifa and Liaquat National Hospital randomly we selected first and moved down until we identified 5 doctors and 16 paramedical staff for PNS Shifa and 6 doctors and 25 paramedical staff for Liaquat National hospital.

After ethical review committee of Dow University of Health Sciences (DUHS) approval, data collection started. Principal investigator explained the nature and purpose of study to all selected participants. Data were collected from randomly selected health care providers using survey methodology until the sample size was achieved. Confounder was managed through randomized selection of subjects. Confidentiality and anonymity was maintained to obtain as frank answers as possible. To avoid non response bias, questionnaire used (Global adult tobacco survey collaborative group, 2011) was not too long and did not take much time to complete. The questionnaire comprised of three parts. Part A was designed to measure socio-demographic data including age, gender, type of health care provider, department, specialty, duration of occupation. Part B was about the smoking status. Part C determined the socio demographic, environmental and personal factors, which could contribute in the continuation of current smoking among health care providers. The factors that were assessed were influence of friend/peer pressure, addiction, lack of will power and lack of incentive which have previously been identified as significantly associated with smoking among health care providers (Yan et al.,2008). The operational definition for these factors was taken as:

Lack of will power: It is the inability to try to quit smoking ever in the past.

Lack of incentive: Lack of due promotions and increments in salary despite putting in efforts.

Influence of friends and peer pressure: If a person spends more than 6hours (25% of the day) in the company of those friends or colleagues who smoke and if the majority (>50%) of his friends are smokers he is influenced by them.

Addiction: A person is addicted if he smokes even at workplace and home and the absence of smoking causes inability to continue routine work.

SPSS v. 20.0 was used to enter and analyze the data. Categorical variables were presented as frequencies with percentages. Mean and standard deviation was computed for continuous variable i.e. age. Chi-Square test of association was used to see effect of demographic variables on factors leading to smoking habits. Odds ratio was computed for estimating the effect within factors. P Value less than 0.05 was considered to show significant effect.

## 3. Results

There were total 116 (64.4%) males and 64 (35.6%) females. Most of them (n = 128, 71.1%) were from public university (DUHS). Forty three were doctors, 67 nurses and 70 were technicians. Fifty five percent were working in medicine department. The remaining 45% (n = 81) were employees of surgery department.

Fifty two participants were smokers for the past one year (28.9%). Among them, 21 (11.7%) smoked more than 5 cigarettes per day. (Table 1)

**Table 1:** Shows the frequency of different variables among the sample population

		Frequency	Percent
Gender	male	116	64.4
	female	64	35.6
Hospital/Institute	DUHS	128	71.1
	Shifa	21	11.7
	liaquat	31	17.2
Type of Health care provider	doctor	43	23.9
	nurse	67	37.2
	technician	70	38.9
Department	medicine	99	55
	surgery	81	45
Are you a smoker for the past one year?	yes	52	28.9
	no	128	71.1
Number of cigarettes that you smoke per day	less than 5	31	59.6
	more than 5	21	40.4
How many of your friends or colleagues smoke?	less than 50%	30	57.7
	more than 50%	22	42.3
How many hours do you spend in the company of your smoker friends?	less than 6 hrs	30	57.7
	more than 6 hrs	22	42.3
Influence of friends and peer pressure	Not Influenced	32	61.5
	Influenced	20	38.5
Do you smoke at work place?	no	18	34.6
	yes	34	65.4
Do you smoke at home?	no	17	32.7
	yes	35	67.3
Are you able to continue routine work in the absence of smoking?	yes	21	40.4
	no	31	59.6
Addiction	Not Addicted	31	59.6
	Addicted	21	40.4
Lack of will power	no	23	44.2
	yes	29	55.8
Are you getting your promotion on time?	yes	35	67.3
	no	17	32.7
Are you getting your due increment in salary?	yes	29	55.8
	no	23	44.2
Lack of incentive	No	36	69.2
	Yes	16	30.8

Twenty two (12.2%) participants had more than fifty percent smoker friends. The frequency of participants spending hours with such friends for more than 6 hours was same (n = 22, 12.2%). Though, 20 (11.1%) were found to smoke due to peer influenced as described in our operational definition.

At workplace, 18.9% (n = 34) participants smoked while 35 (19.4%) participants reported to smoke at home. Thirty one (17.2%) participants acknowledged that they were not able continue work in the absence of smoking. Though, only 21 (11.7%) of them were found to be addicted with smoking.

Twenty nine participants (16.1%) were unable to quit smoking ever and hence found to have lack of will power. Seventeen (9.4%) participants were not getting their promotion on time and 23 (12.8%) had no due salary increment. Sixteen (8.9%) ensued lack of incentive in their organization. (Table 1)

There was no leading significant effect of demographic variables on smoking habit and influencing factor for the same. (Table 2)

**Table 2:** Depicts the effect of demographic variables on smoking habit

		Gender		P Value	Hospital/Institute			P Value	Type of Health care provider			P Value	Department		P Value
		male	female		DUHS	Shifa	liaquat		doctor	nurse	technician		medicine	surgery	
Are you a smoker for the past one year?	yes	45	7	<0.001	32	12	8	0.0098	11	17	24	0.444	30	22	0.78
		86.54%	13.20%		60.40%	22.60%	15.38%		20.80%	32.10%	46.15%		56.60%	42.31%	
	No	71	57		96	9	23		32	50	46		69	59	
		55.47%	44.90%		75%	7.10%	17.97%		25.20%	39.40%	35.94%		54.30%	46.09%	
Number of cigarettes that you smoke per day	less than 5	24	7	0.033	22	3	6		5	11	15	0.554	18	13	0.947
		77.40%	22.60%		71.00%	9.70%	19.40%		16.10%	35.50%	48.40%		58.10%	41.90%	
	more than 5	21	0		10	9	2		6	6	9		12	9	
		100.00%	0.00%		47.60%	42.90%	9.50%		28.60%	28.60%	42.90%		57.10%	42.90%	
Influence of friends and peer pressure	Not Influenced	25	7	0.035	26	0	6		7	10	15	0.959	18	14	0.79
		78.10%	21.90%		81.30%	0.00%	18.80%		21.90%	31.30%	46.90%		56.30%	43.80%	
	Influenced	20	0		6	12	2		4	7	9		12	8	
		100.00%	0.00%		30.00%	60.00%	10.00%		20.00%	35.00%	45.00%		60.00%	40.00%	
Addiction	Not Addicted	25	6	0.219	23	3	5		5	9	17	0.289	21	10	0.075
		80.60%	19.40%		74.20%	9.70%	16.10%		16.10%	29.00%	54.80%		67.70%	32.30%	
	Addicted	20	1		9	9	3		6	8	7		9	12	
		95.20%	4.80%		42.90%	42.90%	14.30%		28.60%	38.10%	33.30%		42.90%	57.10%	
Lack of will power	No	19	4	0.686	16	3	4		7	5	11	0.2	12	11	0.473
		82.60%	17.40%		69.60%	13.00%	17.40%		30.40%	21.70%	47.80%		52.20%	47.80%	
	yes	26	3		16	9	4		4	12	13		18	11	
		89.70%	10.30%		55.20%	31.00%	13.80%		13.80%	41.40%	44.80%		62.10%	37.90%	
Lack of incentive	No	33	3	0.182	20	10	6		9	13	14	0.276	20	16	0.64
		91.70%	8.30%		55.60%	27.80%	16.70%		25.00%	36.10%	38.90%		55.60%	44.40%	
	Yes	12	4		12	2	2		2	4	10		10	6	
		75.00%	25.00%		75.00%	12.50%	12.50%		12.50%	25.00%	62.50%		62.50%	37.50%	

It was found that those who were influenced by their peers were 8.33 times more prone to be addicted to smoking than those who were less influenced. Similarly, the likelihood of addiction increased up to 76.9% with the lack of incentives causes. None of the other factors were significantly associated with each other. (Table 3)

**Table 3:** Shows the relation between peer pressure, addiction, lack of will power and incentive

		Addiction		P Value	Lack of will power		P Value	Lack of incentive		P Value
		Not Addicted	Addicted		no	yes		No	Yes	
Influence of friends and peer pressure	Not Influenced	25	7	0.001	16	16	0.289	21	11	0.476
		78.10%	21.90%	OR=8.33 (95%CI: 2.34-29.72)	50.00%	50.00%	OR=1.86 (95%CI: 0.59-5.87)	65.60%	34.40%	OR=0.64 (95%CI: 0.18-2.22)
	Influenced	6	14		7	13		15	5	
		30.00%	70.00%		35.00%	65.00%		75.00%	25.00%
Addiction	Not Addicted				13	18	0.686	18	13	0.034
					41.90%	58.10%	OR=0.79 (95%CI: 0.26-2.42)	58.10%	41.90%	OR=0.23 (95%CI: 0.06-0.95)
	Addicted				10	11	18	3
					47.60%	52.40%	85.70%	14.30%
Lack of will power	no							17	6	0.515
								73.90%	26.10%	OR=1.49 (95%CI: 0.45-4.98)
	yes							19	10
								65.50%	34.50%

## 4. Discussion

In Lahore, Pakistan the average total prevalence of smoking amongst health care providers, which includes doctors and paramedic staff, is 37.18% and 35.74% respectively with a male to female gender prevalence being 50.31% to 7.04% amongst doctors and 61.53% to 2.22% amongst paramedic staff (Malick et al.,2010). In a research carried out in the neighboring country of Pakistan (China), amongst all the respondents sampled, 20.8% were found to be current smokers who represented about 35.7% of the physician doctors and 1.4% of the nurses (Yan et al.,2008). This is in comparison to our research which establishes that out of the 180 people sampled, 28.9% people smoked with 25 % being male smokers and only 3.9% being female smokers. Thus it can be stated that our data follows the regional trend, as per the gender aspect, showing a low frequency of female health care provider smokers and a moderate to high frequency of male smokers in our part of the region of Asia. This, as opposed to Italy which shows a higher percentage of smoking amid the female members, 41% of the hospital staff as compared to males (Zanetti et al.,1998). However, the exact reason for such a region wise change is yet unknown. Our research also shows an increasing trend of smoking from doctors (6.1%) to nurses (9.4%) and technicians (13.9%) which is opposed to a research in China which shows a high prevalence of smoking amongst doctors and a low prevalence amongst nurses (Yan et al.,2008). The lower level of smoking amongst doctors, as indicated by our research, is supported by a research in Poland (Cofta & Staszewski, 2008).

Of the total people who do smoke, our research established that 40.4% of the people smoked more than 5 cigarettes per day. This is consistent with the Pakistani trend supported by the fact that about 26.44% doctors and 41.89% paramedics smoked 11-20 cigarettes a day (Malick et al.,2010). Furthermore, 65.4% of smokers also smoked in their work place. Therefore, it can be stated that of the hospital staff who smoke, a much significant proportion are more prone towards smoking in their work place, which could be a representative measure of the level of addiction and tolerance. Our research also laid down strong association of smoking with peer pressure and the attitude of friends as shown by the fact that of the total people who smoked, 42.3% had more than 50% friends who smoked and spent more than 6 hours in the presence of their smoking friend company. This is supported by a number of researches at both, medical student and hospital level. In fact a research established an independent association of smoking and peer pressure amongst medical students (Ganesh Kumar, Subba, Unnikrishna, Jain, & Badiger, 2011) and another research highlighted its strong role amongst 14 -17 year old teenagers (Husain et al.,2012). This is further pressed upon by a research which found the role of peer pressure in smoking in 83.6% of the future physicians out of which 42% had addictions and was one of the most important risk factor (57.69%) for the initiation of the habit of smoking (Basu et al.,2011). The dominating role of peer pressure was also a strong determinant amongst doctors and paramedic staff, supported by a research which stated that 55.17% doctors and 75.68% paramedics started smoking under influence of friends. This is further supported by a research which found that 31% of the hospital staff was smoking under influence of peer pressure (Chaudhry, Chaudhry, & Mamood, 2009). Association of peer pressure with smoking is following an increasing trend and therefore a strong relationship between peers’ habbitsand an individual susceptibility towards smoking exists. Therefore steps taken to reduce peer pressure can significantly help reduce the incidence of smoking. In terms of the level of addiction, our data reveals a high level of addiction shown by the fact that of the total people who smoked, 65.4% smoked at the work place which includes hospitals. This can be used as an indicator showing the level of addiction amongst hospital staff which is further supported by the fact that 59.6% of the people couldn’t carry out their routine work without smoking. Therefore, it is vital that important steps must be taken to make hospitals completely smoke free to aid healing of patients.

The exact cause of smoking is debatable but our statistics demonstrated that inadequate salaries and lack of promotion opportunities may also have a role in smoking. The reason that could be hypothesized is that such factors play a role in up regulating the level of anxiety amongst health care providers who then resort to smoking initially as an escape and then end up in being addicted. In a developing country like Pakistan, reasons other than monetary issues like political instability and violence could be amongst contributing factors towards smoking. Other perpetuating factors could be increased workload, which may be both psycho-social and physical in nature (Zysnarska, Bernad, Adamek, & Maksymiuk, 2008). It is therefore important that such issues must be solved at both hospital and government level so as to ensure and maintain appropriate levels of hospital functioning which would ultimately aid the patient’s needs. It can also be hypothesized that if such factors are overlooked, they could yield devastating results in future, with the health care providers then resorting to more harmful substances of abuse and eventually culminating in disaster of the profession.

The moderately high prevalence of smoking amongst health care providers is alarming as they are setting a bad example to patients by being uncritical to this habit by rarely asking patients about their smoking and rarely advising them not to smoke (Stojanović et al., 2013). This is further elaborated by a research which found out that of the physicians who smoke, 25% do not warn their patients about the risks of smoking and about 22% not always give advice to them about quitting (Araya et al.,2012). Therefore strong steps must be taken to decrease it amongst health care providers to indirectly reduce its prevalence in the general population. This could be achieved by early identification of smokers. Therefore researches like these must be carried out at medical school level to identify smokers at an early stage. Schools must devise rules and policiesto discourage smoking (Ghimire, Sharma, Niraula, Devkota, & Pradhan, 2013). The exact causes of smoking must be identified and then eradicated. The government can also play its part by imposing heavy taxation on cigarette to limit its availability and restricting cigarette advertisements. Moreover, motivation by friends and family members could also help. Other researches so as to percept the risk of smoking amongst the general population and health care providers could also be carried out (Power, Neilson, & Perry, 2004). Important steps must be taken so as to promote smoke free environmental policies as it could help reduce the levels of smoking in hospitals and also amongst hospital staff as supported by a research which shows that after such implementations the level of smoking in staff could be reduced by 44% in a 2 year period (Poder, Carroll, Wallace, & Hua, 2012). In fact the ban on smoking in public places has shown that amongst nurses 68% had decreased their tobacco consumption in their working hours and about 28 % had reduced their overall daily consumption (Maurel-Donnarel, Baumstarck-Barrau, Barlesi, & Lehucher-Michel, 2010). To our knowledge this is amongst the few researches which cover such an important aspect in our region.

Our research only focused on doctors, nurses and technicians. It should be noted, however, that inclusion of other members of paramedic staff and dentists in the sample population may have given a better picture.

The doctors could have been further subdivided according to the departments, so as to highlight the department which is involved more in smoking. The sample size of 180 people was very small and couldhave been increased by including more tertiary care hospitals. An increase in sample size increases the reliability of study and the study becomes more representative of the targeted population. A much more validated scale for the measurement of addiction and stress like the Fagerstorm Test could have been used to make the results more valid and consistent. Much more detailed correlates to the multiple causes of smoking, other than stress and promotion, could have been pinpointed so as to identify the correct and exact cause. Furthermore it could have been of great use if attitude of doctors and paramedics towards smoking cessation was asked.

## 5. Conclusion

Our results clearly indicate that a large number of health care providers smoke which should be a serious concern. We also found that addiction was significantly associated with lack of incentives and peer pressure. Hence our health agencies should take immediate action in order to curtail the heaving burden of smoking and its related health consequences.
